# Supplementary material for: Metamaterial assisted illumination nanoscopy via random super-resolution speckles
Source: Nat Commun. 2021 Mar 10;12:1559. doi: 10.1038/s41467-021-21835-8 (PMC7946936; doi:10.1038/s41467-021-21835-8)
Supplement: Supplementary file 1 — Supplementary Information [file 41467_2021_21835_MOESM1_ESM.pdf]

Supporting Information

Metamaterial assisted illumination nanoscopy via random super-resolution speckles

Yeon Ui Lee[1,‡], Junxiang Zhao[1, ‡], Qian Ma[1,‡], Larousse Khosravi Khorashad[1], Clara Posner[2], Guangru Li[1], G. Bimananda M. Wisna[3], Zachary Burns[1], Jin Zhang[2], Zhaowei Liu[1,3,4]*

[1] *Department of Electrical and Computer Engineering, University of California, San Diego, 9500 Gilman Drive, La Jolla, California 92093, USA*

[2] *Department of Pharmacology, University of California San Diego, 9500 Gilman Drive, La Jolla, California 92093, USA*

[3] *Material Science and Engineering Program, University of California, San Diego, 9500 Gilman Drive, La Jolla, California 92093, USA*

[4] *Center for Memory and Recording Research, University of California, San Diego, 9500 Gilman Drive, La Jolla, California 92093, USA*

‡ These authors contributed equally to this work

*Corresponding author email address: zhaowei@ucsd.edu

Contents

S1. Transmittance of Ag-SiO_2 multilayer with different silver layer thickness

S2. Fourier transform of speckles 10 nm away from HMM top surface

S3. Normalized cross-correlation between speckles at different incident angles

S4. Speckle-MAIN experimental setup

S5. Super-resolution image of fluorescent beads with 0.8 NA and 80 sub-frames

S6. Correlation length measured from AFM heights mapping

S7. Simulated model of the rough surface and correlation length

S8. Penetration depth with respect to different illumination k

S9. *z*-location dependence of high-resolution speckle in speckle-MAIN

S10. Blind-SIM, SOFI, ESI, and MUSICAL reconstruction results

S11. FRC Resolution for speckle-MAIN with blind-SIM reconstruction

S12. SEM images and speckle-MAIN images

S1. Transmittance of Ag-SiO_2 multilayer with different silver layer thickness.

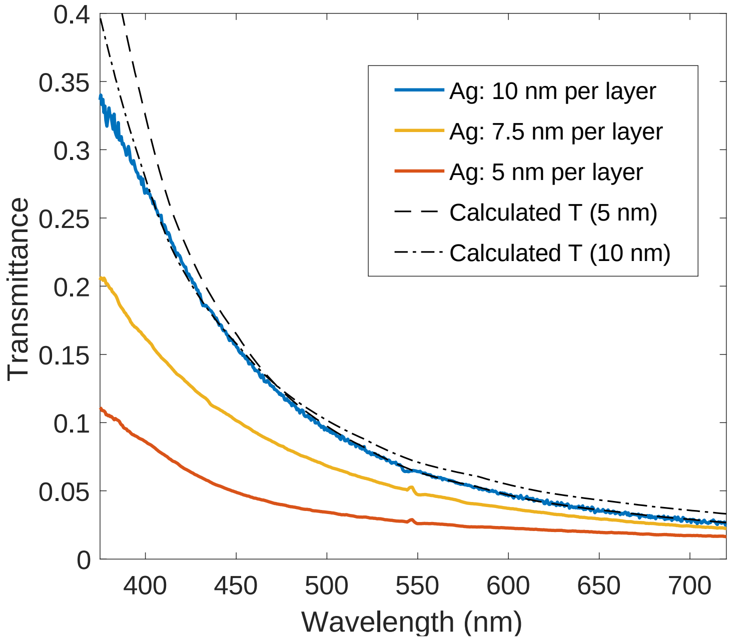

Supplementary Figure 1. Transmittance of Ag-SiO_2 multilayer with different silver layer thickness. Blue solid: measured transmittance of 3 pairs of 10 nm Ag and 4nm SiO_2. Yellow solid: measured transmittance of 4 pairs of 7.5 nm Ag and 3 nm SiO_2. Red solid: measured 6 pairs of 5 nm Ag and 2 nm SiO_2. Black dashed: calculated transmission of 6 pairs of 5 nm Ag and 2 nm SiO_2; Black dot-dashed: calculated transmission of 3 pairs of 10 nm Ag and 4 nm SiO_2.

Multilayer HMM preparation. Ag-SiO_2 multilayer, composed of 3 pairs of 10 nm Ag and 4 nm SiO_2 layers, were prepared by alternately DC magnetron sputtering and RF magnetron sputtering at room temperature. Sputtering rates for Ag and SiO_2 at 200 W were ~1 nm/s and ~0.8 nm/min, respectively. The pressure of the chamber was 2.5×10^{-6} mtorr and the Ar pressure was at 3.2 mtorr for Ag and 5 mtorr for SiO_2 during the deposition. The glass substrate (VMW Micro cover glasses, No.1 1/2) is cleaned by Acetone, IPA, and water several times and dried by compressed air. An adhesion layer (<1 nm thickness) of Cr is deposited on the substrate prior to the deposition of Ag-SiO_2 multilayer. The sputtering machine is Denton discovery 635 at Nano3 facility at Calit2, UCSD.

S2. Fourier transform of speckles 10 nm away from HMM top surface

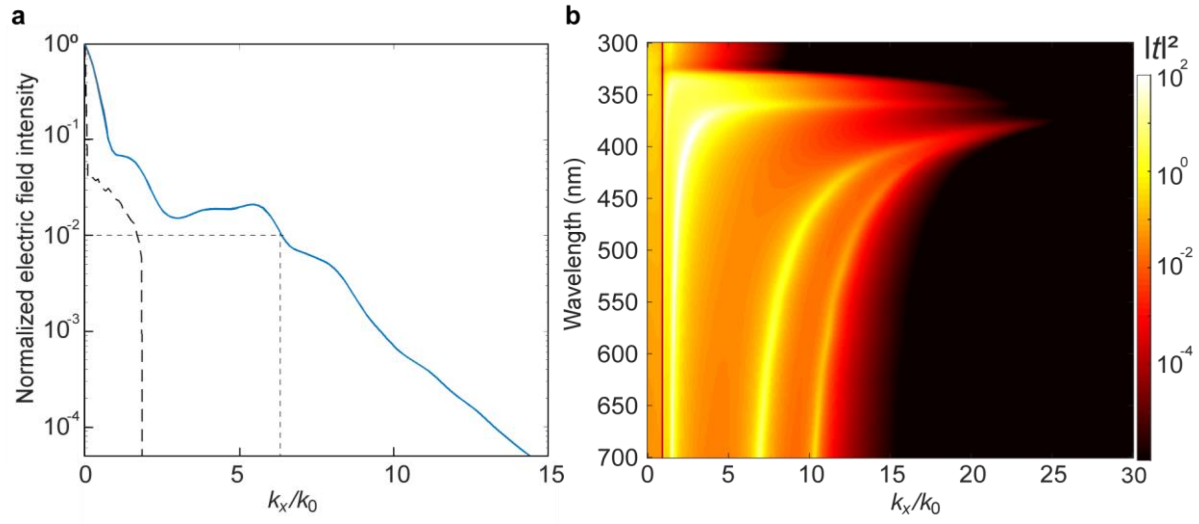

Supplementary Figure 2. a Fourier transform of speckles 10 nm away from HMM (3 pairs of 10 nm Ag and 4 nm SiO_2 on glass) at λ = 500 nm. k_x stands for the spatial frequency of Fourier transform of intensity patterns. k_x is normalized to the free space wave vector k_0. The black dashed line indicates the Fourier transform of speckles on glass. **b** Calculated optical transfer function (OTF, transmission, $|t|^2$) as a function of k_x/k_0.

S3. Normalized cross-correlation between speckles at different incident angles

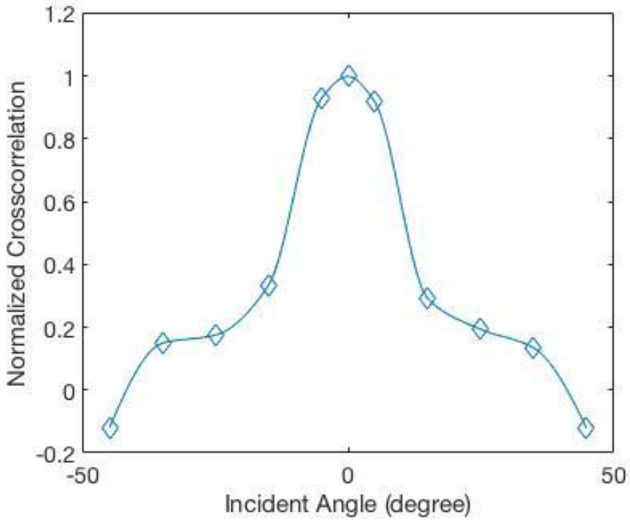

Supplementary Figure 3. Normalized cross-correlation between speckles at different incident angles. The HMM (3 pairs of 10 nm Ag and 4 nm SiO_2 on glass) was used.

S4. Speckle-MAIN experimental setup

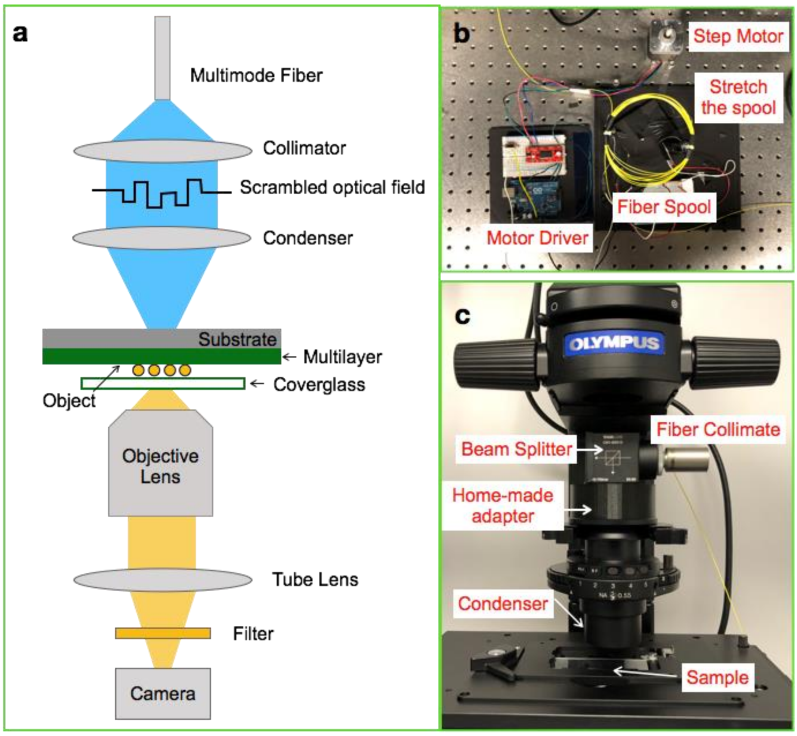

Supplementary Figure 4. Speckle-MAIN experimental setup. a Sketch of essential optical components. **b** Speckle Generator based on multimode fiber. A laser beam is guided by the multimode

fiber to an inverted microscope. The multimode fiber spool (tens of loops) is stretched by a step motor to change the output electrical field. **c** Home-made adapters to an inverted microscope condenser. The adapter, together with the multi-mode fiber system, and multilayer-coated sample substrate, converts the epi-fluorescent microscope into speckle-MAIN.

S5. Super-resolution image of fluorescent beads with 0.8 NA and 80 sub-frames

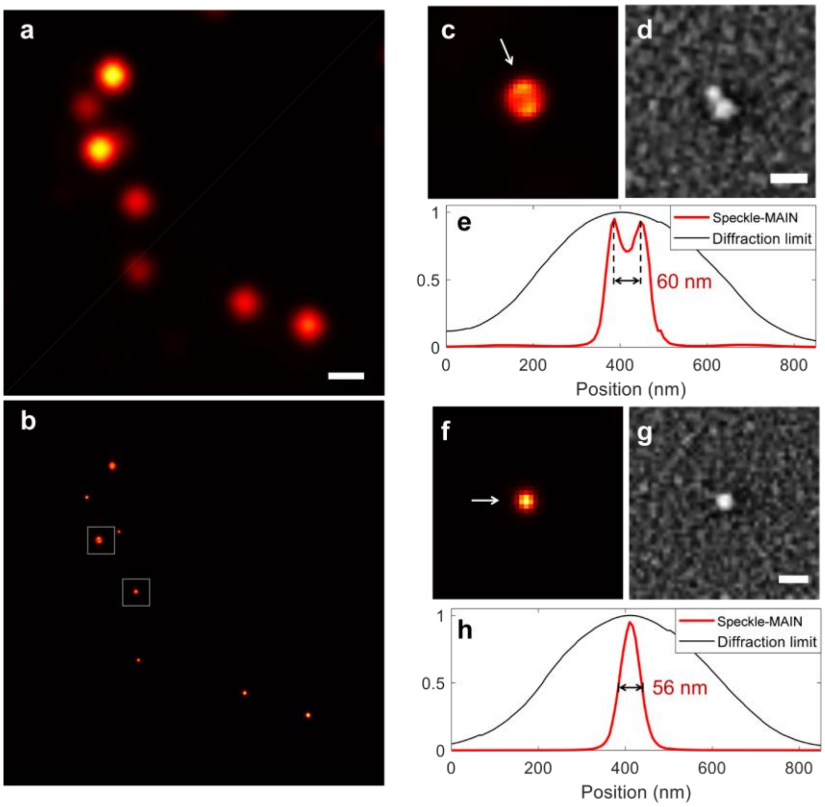

Supplementary Figure 5. Speckle-MAIN super-resolution image of fluorescent beads with 0.8 NA and 80 sub-frames. a Diffraction-limit Image. Scale bar: 600 nm. Objective lens: Zeiss 50×/0.8. **b** Reconstructed Image. Number of raw camera frames: 80; Illumination wavelength: 450–480 nm. Step: 10 nm; Bandwidth: 10 nm. For each wavelength channel, 20 frames are acquired by changing the scrambled incident optical field. **c-h** zoom in images of (b) at indicated location. **d,g** SEM images of fluorescent beads at indicated locations. Scale Bar: 100 nm. **e,h** Cross-section (normalized intensity) of image (c,f) along indicated direction.

S6. Correlation length measured from AFM heights mapping

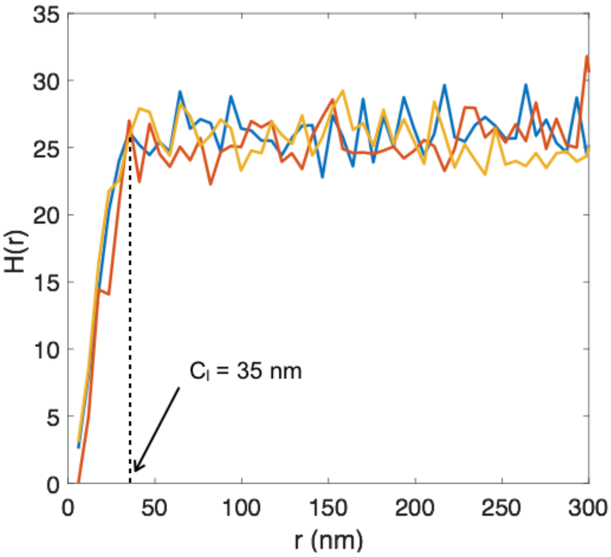

Supplementary Figure 6. Correlation length (C_l) measured from AFM heights (H(*r*)) mapping.

S7. Simulated model of the rough surface with RMS and correlation length

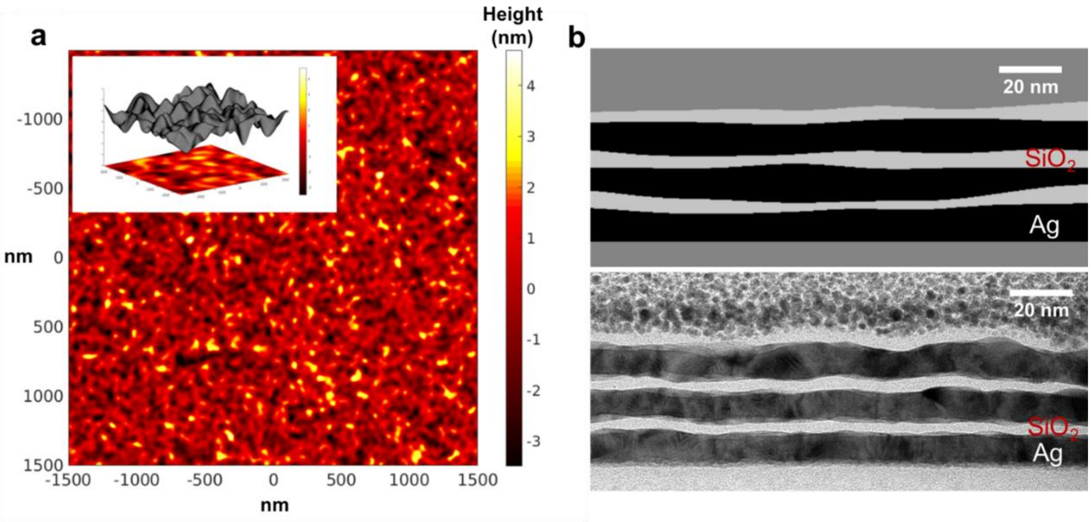

Supplementary Figure 7. Simulated model of the rough surface with RMS = 1.1 nm and correlation length of ~35 nm. a The simulated top morphology of a rough interface between Ag and SiO_2. Inset: 3D drawing of the simulated interface; **b** The side view of the simulated model compared to TEM measurement.

S8. Penetration depth with respect to different illumination k

Speckle-MAIN takes advantage of ultra-high-resolution speckle that is strongly confined to the material interface to achieve super-resolution. The penetration depth of the illumination pattern is thus determined by the momentum matching condition on HMM surface. With high-k speckle illumination pattern propagating on the HMM surface, the wavevector in air must satisfy the boundary condition for its lateral component $k_{x,\mathrm{air}} = k_{x,\mathrm{HMM}}$. Therefore, the transverse wavevector can be calculated by

$$k_{z,\mathrm{air}} = \sqrt{(n_{\mathrm{air}}k_0)^2 - k_{x,\mathrm{HMM}}^2} \tag{S1}$$

The electric field in air thus exponentially decays away from the interface.

$$E_{\mathrm{air}} = E_0 \exp\left(-ik_{z,\mathrm{air}}z\right) \tag{S2}$$

The intensity of the evanescent wave decays with the square of the electric field, so at a distance z, the intensity has decreased by a factor of $\exp\left(-2ik_{z,\mathrm{air}}z\right)$. The penetration depth $\delta = -1/2ik_{z,\mathrm{air}}$ is defined as the distance for the intensity to decay by a factor of 1/e.

Figure S8 shows the penetration depth with respect to different illumination k-vector. The penetration depth of the illumination is sacrificed for the high-k illuminations (high resolution speckles) while the traditional low-k illuminations (k_x,<NA×k_0) keep a deep penetration depth.

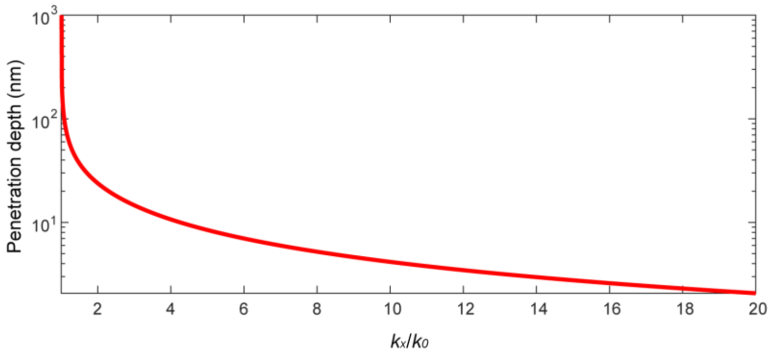

Supplementary Figure 8. Penetration depth with respect to different illumination *k*.

S9. z-location dependence of high-resolution speckle in speckle-MAIN

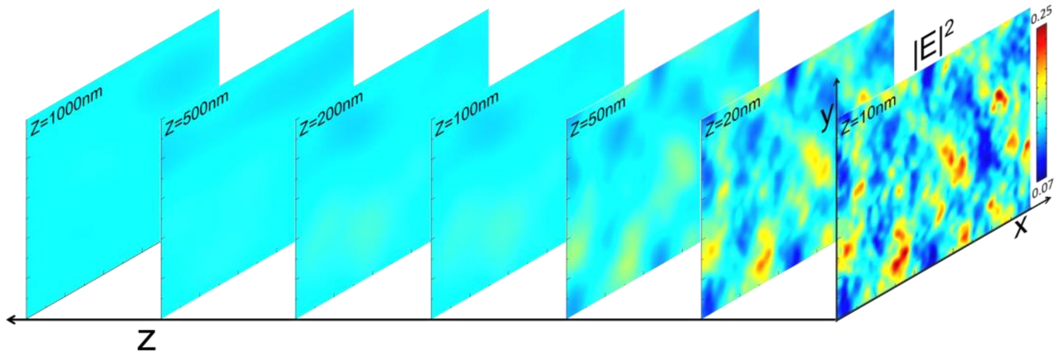

Supplementary Figure 9. z-location dependence of high-resolution speckle in speckle-MAIN. FDTD calculated near field speckle intensity distributions are shown at 10 nm $\leq z \leq$ 1000 nm. The windows are 1 $\mu m \times 1$ μm. The HMM (3 pairs of 10 nm Ag and 4 nm SiO_2) located at z = 0 was used for the simulation. The maps are simulated for normal incident beam ($\theta = 0°$) at wavelength $\lambda = 500$ nm. For comparison convenience, we showed all the intensity maps at the same color scale.

S10. Blind-SIM, SOFI, ESI, and MUSICAL reconstruction results

Speckle-MAIN provides a platform to generate ultra-high-resolution speckle illumination and should in principle work with all speckle based super-resolution reconstruction methods including blind-SIM, speckle-SOFI, ESI, and MUSICAL. However, each reconstruction algorithm is based on a different mathematical method and behaves differently when trying to reconstruct a high-resolution image as shown in Fig. S10.

SOFI utilizes the blinking statistics to achieve super-resolution. While SOFI is the least computationally taxing method and generates low amount artefacts, it cannot achieve very high resolution. The resolution improvement of SOFI is $\sqrt{N}$, where N is the cumulant order. Due to extremely high contrast in reconstruction using high order cumulant, we choose cumulant order to be less than 8 for practical reconstruction. ESI calculates the Shannon-Entropy of intensity fluctuations of fluorophores and requires high entropy calculation iterations (>3) and high

centralized moment order. However, high ESI iterations will cause extreme contrast similar to SOFI and high moment order will pixilate the image when reconstruction bandwidth becomes larger than Nyquist-limit. MUSICAL generates super-resolution images by calculating eigenimages with corresponding eigenvalues and using an indication function to plot out the location of each individual emitters. MUSICAL can achieve resolution similar to single molecule localization methods when the number of fluorophores within a small area is smaller than either the number of sub-pixels in this area or the number of sub-frames, as in this case the signal space and null space are well defined. However, when there are too many emitters in the field of view, a threshold of eigenvalue is chosen to separate signal space and null space. Overall, we studied and compared multiple possible reconstruction algorithms for speckle-MAIN and believed blind-SIM is the most suitable reconstruction method for the majority of cases.

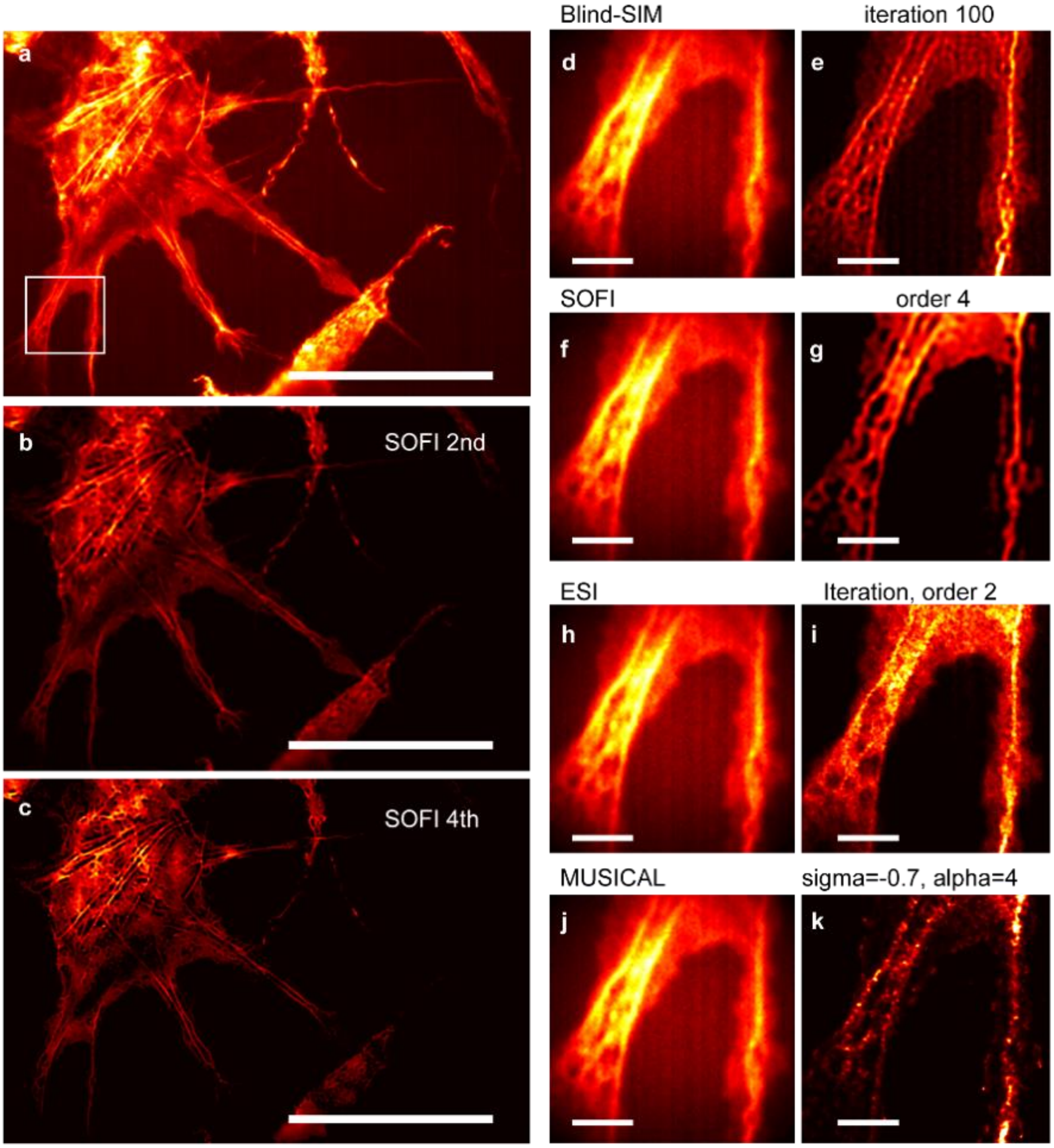

Supplementary Figure 10. Blind-SIM[1], SOFI[2], ESI[3], and MUSICAL[4] reconstruction results. a Diffraction limited image. Scale bar is 20 µm. **b** SOFI reconstruction with second-order **c** fourth-order. **d, f, h, j** Zoom-in-view of the white box area in (**a**). **e** Blind-SIM reconstruction with iteration 100. **g** SOFI reconstruction. **i** ESI reconstruction results with second-order. **k** MUSICAL reconstruction. Scale bar is 2 µm.

S11. FRC Resolution for speckle-MAIN with blind-SIM reconstruction

Fourier ring correlation (FRC) resolution criteria has become a widely accepted resolution criteria for localization based super-resolution microscopy. FRC requires two reconstructed images based on two independent subsets of data and computes the statistical correlation of the Fourier components of the two images on the perimeter of circles of constant spatial frequencies.

We separate the raw data set to two statistically independent subsets each containing 150 sub-images and performed blind-SIM reconstruction separately. The FRC curve is calculated with the equation:

$$\mathrm{FRC}(q) = \frac{\sum_{\vec{q}\in \mathrm{circle}} \widehat{f_1}(\vec{q})\widehat{f_2}(\vec{q})^*}{\sqrt{\sum_{\vec{q}\in \mathrm{circle}} \left|\widehat{f_1}(\vec{q})\right|^2}\sqrt{\sum_{\vec{q}\in \mathrm{circle}} \left|\widehat{f_2}(\vec{q})\right|^2}} \tag{S3}$$

where $\vec{q}$ and $\hat{f}$ denotes spatial frequencies, $\widehat{f_1}$ and $\widehat{f_2}$ are the Fourier transform of the two reconstructed images. Figure S11 (c) shows the FRC curve for beads. The standard 1/7 FRC resolution criteria illustrates 43 nm Fourier space cutoff with speckle-MAIN. Fig. S11 (f) and Fig. S11 (i) show the FRC curve for Qdot 605 and cells, respectively. The standard 1/7 FRC resolution criteria illustrates 65 nm and 68 nm Fourier space cutoff achieved with speckle-MAIN. However, although FRC can be used as a resolution criterion for blind image reconstruction algorithm, the reported FRC resolution value is typically lower than the highest achievable resolution reconstructed with full data set. From an information theory point of view, at least αN^2 sub-frames are required to achieve an N-fold resolution improvement. α is the oversampling factor. Since the number of sub-frames is halved for each independent set during FRC calculation, the maximum achievable resolution is lowered as it's less likely to find an optimal solution for high-k information which has low power density. Nevertheless, FRC still provides a convincing resolution measurement for speckle-MAIN albeit being slightly lower than maximum possible resolution value.

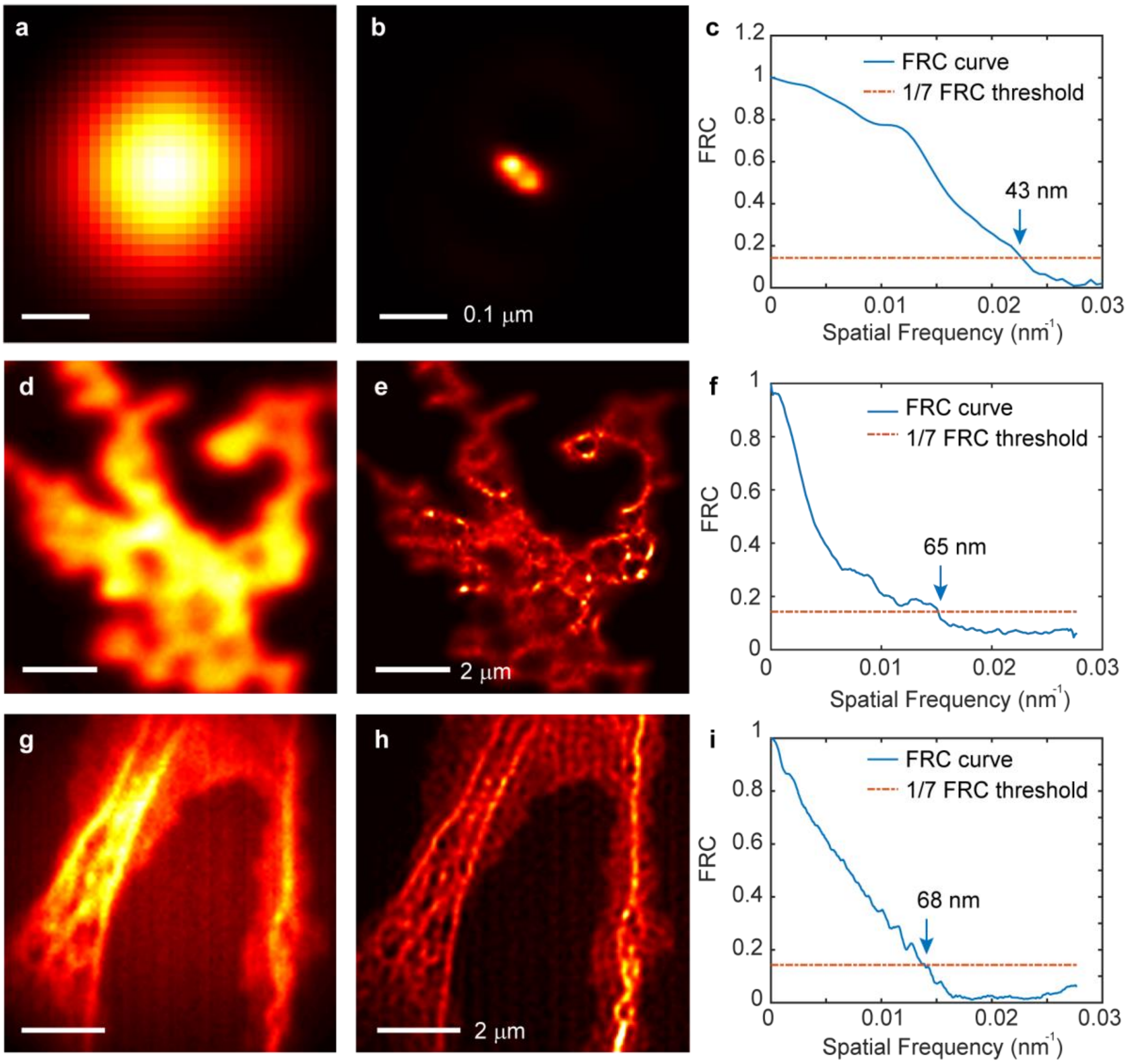

Supplementary Figure 11. a Diffraction limited image and **b** reconstructed image of two fluorescence beads (center to center distance 40 nm), **c** FRC resolution criteria curve for the bead image. The standard 1/7 FRC resolution criteria illustrates 43 nm Fourier space cutoff with speckle-MAIN. **d-f** are diffraction limited image, reconstructed image, and FRC curve of the aggregated Q-dot 605 sample, respectively. **g-i** are diffraction limited image, reconstructed image, and FRC curve of the Cos-7 cell sample, respectively.

S12. SEM images and speckle-MAIN images

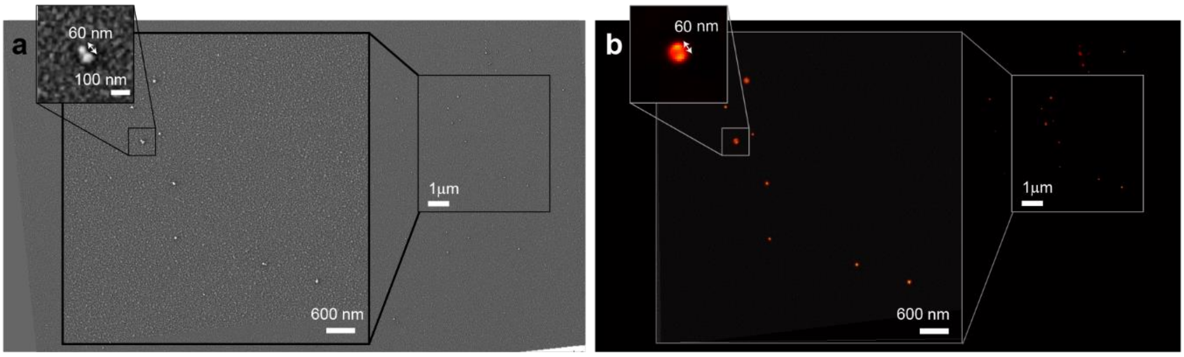

Supplementary Figure 12. a SEM images of fluorescent beads. **b** Reconstructed super-resolution speckle-MAIN image of the fluorescent beads.

Supplementary References

1. Mudry, E. *et al.* Structured illumination microscopy using unknown speckle patterns. *Nat. Photonics* **6**, 312–315 (2012).
2. Dertinger, T., Colyer, R., Iyer, G., Weiss, S. & Enderlein, J. Fast, background-free, 3D super-resolution optical fluctuation imaging (SOFI). *Proc. Natl. Acad. Sci.* **106**, 22287–22292 (2009).
3. Yahiatene, I., Hennig, S., Müller, M. & Huser, T. Entropy-Based Super-Resolution Imaging (ESI): From Disorder to Fine Detail. *ACS Photonics* **2**, 1049–1056 (2015).
4. Agarwal, K. & Macháň, R. Multiple signal classification algorithm for super-resolution fluorescence microscopy. *Nat. Commun.* **7**, 13752 (2016).
